# Kaposi Sarcoma Mimicking a Lingual Lesion in an HIV-Negative Patient: A Case Report

**DOI:** 10.7759/cureus.57131

**Published:** 2024-03-28

**Authors:** Victor Ramon Andrade-Carmona, Lizette Guadalupe Carmona-Araiza, Danny Soria-Cespedes, Laura Gómez-Virgilio, Gustavo López-Toledo

**Affiliations:** 1 Otolaryngology - Head and Neck Surgery, Hospital Angeles Lindavista, Mexico City, MEX; 2 Pathology, Hospital Angeles Lindavista, Mexico City, MEX; 3 Medical Education, Hospital Angeles Lindavista, Mexico City, MEX

**Keywords:** human herpesvirus 8, tonsillectomy, human immunodeficiency virus, oropharyngeal lesions, kaposi sarcoma

## Abstract

Tonsillar Kaposi sarcoma is rare, reported in patients with human immunodeficiency virus (HIV) infection. This case report of a tonsillar Kaposi sarcoma (KS) in an HIV-negative male patient, initially misinterpreted as a lingual lesion diagnosed with KS following tonsillectomy, highlights the value of a differential diagnosis in atypical presentations. The case report discusses the etiologic agent of KS, its detection and treatment, and a few case reports about tonsillar KS with no association with AIDS. The case underscores the diagnostic challenge of oropharyngeal lesions, particularly in patients with risk factors but negative HIV status.

## Introduction

Kaposi sarcoma (KS) is an angioproliferative neoplasm whose aetiologic agent is KS herpesvirus (KSHV) or herpesvirus type 8 (HHV-8). KS was defined by Dr. Moritz Kaposi in 1872, based on many cases of skin multifocal pigmented sarcoma in old European men [1]. KS has been recognized into four epidemiological and clinical subtypes: classic, endemic, iatrogenic, and acquired immune deficiency syndrome (AIDS)-associated. Classic KS mainly presents with clustered papules and nodules in the lower limbs of middle-aged and elderly Mediterranean or Eastern European individuals. In the endemic form of KS, children often present lymphoedema and visceral dissemination, and the adults present lower-limb lesions; this subtype occurs most commonly in sub-Saharan Africa. Iatrogenic KS develops in organ transplant individuals with immunosuppressive therapy. These individuals present cutaneous KS lesions as well as mucosal and visceral disease. AIDS-related KS is growing in individuals who are seropositive for HIV. They exhibit numerous cutaneous lesions on the extremities, torso, and face in addition to mucosal injuries and visceral involvement and can also present tumor-associated edema [2]. A fifth subtype is now accepted: nonepidemic KS in men who have sex with men (MSM), are HIV-negative, and have no other perceived causes for immunodeficiency. In this subtype, the individuals present few lesions in the skin, and visceral and mucosal disease is unusual [3].

KS clinical manifestations, on many occasions, are cutaneous while oral KS (OKS) recurrently occurs in patients with AIDS [4]. However, other types of Kaposi sarcoma lesions are less common, even more so for head and neck cases, and an isolated oropharyngeal location, in particular, the tonsillar of persons negative for HIV, is highly infrequent [5]. An oral lesion at the beginning of KS in an HIV-negative patient is a factor that can lead to an erroneous diagnosis [6,7].

In this report, we present the case of tonsillar KS in a 50-year-old HIV-negative male patient, initially misinterpreted as a lingual lesion, diagnosed with KS following tonsillectomy. The relevance of this case lies in the atypical clinical presentation and final diagnosis of an HIV-negative patient, underscoring the importance of a meticulous diagnostic approach in unconventional oropharyngeal lesions.

## Case presentation

**Figure 1:**
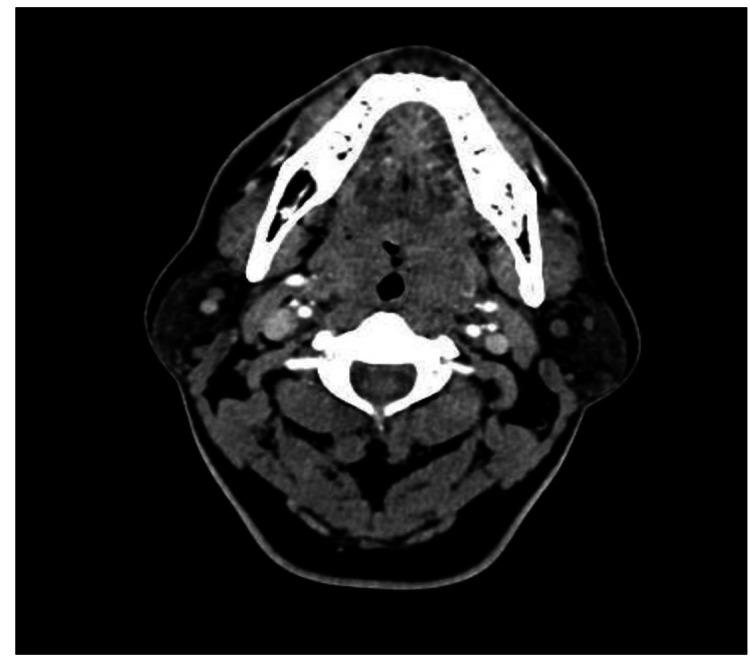
Contrast-enhanced neck CT of the patient Axial contrast-enhanced CT image shows an increase in the size of both tonsils, reducing the airways' light.

A 50-year-old homosexual man with a history of recurrent herpesvirus type 1 infection reported the self-perceived presence of a tongue mass. The patient's initial attempts at self-treatment with mouthwash and manual removal resulted in increased discomfort and bleeding. Subsequent medical consultations resulted in the initiation of antibiotics, with no improvement. A thorough physical examination performed by an otorhinolaryngologist showed an oral cavity with good integumentary and mucosal coloration, well hydrated, adequate dental hygiene, central uvula, preserved lingual mobility, central, mobile trachea, no swallowing problems, and the actual site of the lesion at the base of the left tonsil, not the tongue, characterized by a hard, painful, non-friable mass approximately 1 cm in diameter, was revealed. Contrast-enhanced computed tomography (CT) of the neck suggested a prominent neoplastic process of bilateral tonsillar adenoid tissue, with decreased caliber of the airway, without imaging evidence suggesting abscess or space-occupying lesions, identifying lymph nodes with an inflammatory appearance, with an oval shape and smooth, well-defined margins, which measure less than 10 mm in the short axis, located at levels IB, IIA, IIB, III, IV, and V bilaterally (Figure 1).

Surgical intervention, which included bilateral tonsillectomy, utilized the Crowe-Davis technique using cold dissection and bipolar energy for hemostasis. The process was carried out without incident, leading to a pathological diagnosis of Kaposi's sarcoma.

**Figure 2:**
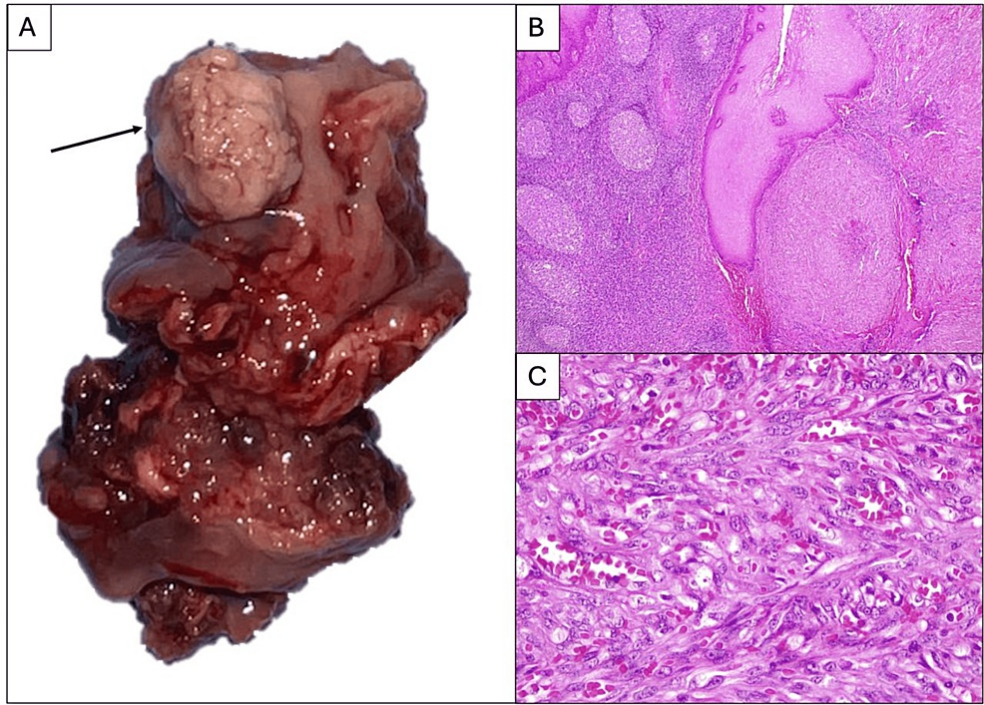
Analysis of the left tonsil at the macroscopic and microscopic levels A. Macroscopic image of the left tonsil features an irregular, light brown neoplastic lesion (1.3 x 0.8 cm) (arrow). B. Hematoxylin-eosin (H&E) staining on the tonsil histological sections shows secondary lymphoid follicles, stratified squamous epithelium, and a spindle-cell malignant neoplastic lesion (magnification x 40). C. The neoplasia is composed of spindle cells with elongated nuclei, vesicular chromatin, small nucleoli, and atypical mitosis with the formation of vascular channels (H&E staining; magnification x 400).

In Pathology, both tonsils were received; the left one showed a 1.3 x 0.8 cm lesion, which was ulcerated, grayish-white, and soft. The cut section revealed a reddish-brown central portion with a grayish-white peripheral area. The proliferation of spindle cells, elongated nuclei, and vesicular chromatin, with atypical mitosis, was identified in the histological sections. Figure 2 shows the resected specimen at the macroscopic and microscopic levels, including H&E staining on the tonsil histological sections where the neoplastic lesion comprises spindle cells with atypical mitosis and from vascular spaces.

**Figure 3:**
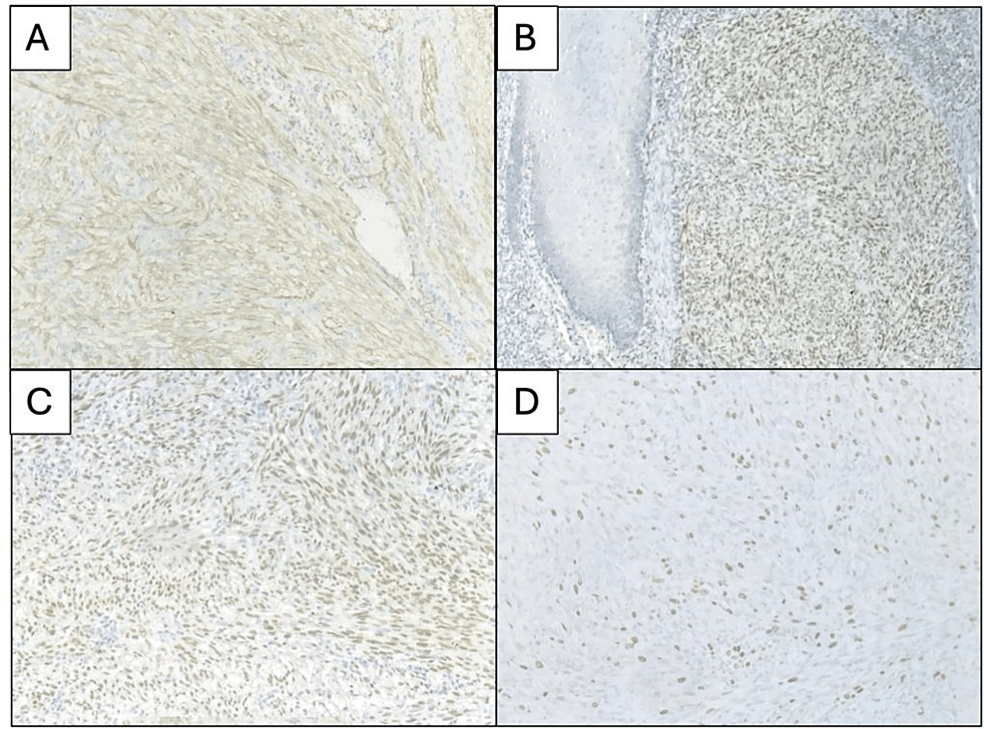
Immunohistochemistry for some markers confirmed Kaposi sarcoma. The neoplastic cells are positive for (A) CD31, (B) ERG, and (C) HHV-8; in addition, (D) these cells have a 30% proliferation index measured with Ki67 (magnification values are different).

The definitive diagnosis was Kaposi sarcoma, confirmed by immunohistochemistry. The neoplastic cells were positive for CD31, ERG, and HHV8 (LANA), with a proliferation index of 30% (measured with Ki67). They were negative for cytokeratin AE1/3, S-100 protein, and desmin. The morphological and immunostaining characteristics correspond to the Kaposi sarcoma diagnosis. Figure 3 illustrates spindle cells immunoreactive for the markers indicated above.

After the diagnosis of Kaposi sarcoma, the patient was investigated for the possibility of HIV infection. HIV testing by enzyme-linked immunosorbent assay (ELISA) and Western Blot returned negative. The patient was referred to infectious disease specialists for further management and remains HIV-negative six months after the surgery.

## Discussion

KSHV is the etiologic agent of KS, an oncogenic virus that manifests with varying disease presentations. This virus may bind and infect several cell types, including endothelial cells. Because KS tumors express endothelial cell markers, endothelial cells are hypothesized to be the KSHV-infected cell type in these tumors [2]. The viral life cycle of KHSV has two phases: latency and lytic replication. In KS, KSHV exists primarily in a state of latency; for this reason, the disease likely takes a more indolent course as compared to HIV-associated or post-transplant KS, where an immunocompromise occurs [8]. Regarding the prevalence of human herpesviruses (HHV) in tonsils, a study reported KSHV was rarely detected in tonsil brushings collected from non-cancerous immunocompetent individuals, with a prevalence of 0.8% in children and 0.7% in adults, showing HHVs like EBV, HHV6B, and HHV7 are frequent in the tonsils of immunocompetent individuals, in contrast with other herpesviruses, like KSHV, that are rarely detected [9].

KSHV can be detected by serologic methods, polymerase chain reaction (PCR), or immunohistochemical methods [10]. In our case, the virus was identified through immunohistochemical examination. Regarding the pathologic diagnosis of KS, hematoxylin and eosin (H&E) staining is used to evaluate many KS features. These features include vascular proliferation in the dermis, an elevated number of vessels without an endothelial cell lining, extravasated blood, and spindle cell proliferation. These spindle cells express endothelial markers; therefore, they are considered KS tumor cells [2]. For us, the immunohistochemical findings were compatible with Kaposi sarcoma.

KS has different treatments depending on the epidemiologic subtype, disease stage, progression, distribution, and immune status [2]. In patients with endemic KS, classic KS, or KS in MSM without HIV infection, treatments directed at the tumors are necessary; for example, surgical excision is generally adequate for localized lesions [7]. In this sense, the clinical approach for treating different KS types is based on small retrospective case series and clinician experience [2].

Head and neck sarcomas have a higher incidence in men than in women (ratio of 2:1) and occur around 50-55 years of age. In most cases, the patient is asymptomatic or presents with a painless neck mass [11]. Imaging diagnosis is essential for diagnostic and staging purposes and helps the surgeon plan therapy. A combination of MRI and CT scanning is preferred on many occasions [12]. KS of the head and neck is rare. In this sense, extensive epidemiological studies have reported the epidemiology of head and neck sarcomas, where the Kaposi sarcoma represents 20-25% of all head and neck sarcomas [12,13]. Besides, an extensive literature review identified 251 published cases of head and neck KS; it indicated the most typical sites of presentation: the palate (hard and soft) and the oropharynx (including the tonsillar fossae) [14]. Regarding the subtype KS, head and neck KS is atypical in non-AIDS cases of KS, with a percentage of <5% of KS cases.

In the literature, there are few case reports about tonsillar KS with no association with AIDS [5,7,10,15]. These case reports described four males and one female with tonsillar KS HIV-negative. The age range was 42-54 years (median: 49 years), and neither patient referred to homosexual activity. We present the case of a tonsillar KS in a 50-year-old, HIV-negative, homosexual male patient who could be classified as the clinical subtype of nonepidemic KS in MSM. On the other hand, the results of HHV-8 immunostaining in our patient suggest a viral causation for oropharyngeal KS. Regarding human herpesvirus type 8 infection in MSM, this has been associated with sexual risk factors, in addition to considering the role of saliva in herpesvirus transmission [16]. In this context, a study reported that in individuals with and without KS and with and without HIV, the prevalence and HHV-8 copies were higher in saliva compared with semen, in addition to suggesting that KSHV can replicate in the oropharynx and that salivary contact could contribute to KSHV transmission [17].

## Conclusions

In conclusion, Kaposi sarcoma, typically associated with HIV infection, can occur in HIV-negative patients, posing diagnostic and therapeutic challenges. It is important to consider differential diagnoses in oropharyngeal lesions, like a unilateral growth of the tonsil, even in patients without positive HIV serology. This case emphasizes the need for a multidisciplinary approach to diagnose and manage atypical oropharyngeal neoplasms.
